# Cross-Entropy as a Metric for the Robustness of Drone Swarms

**DOI:** 10.3390/e22060597

**Published:** 2020-05-27

**Authors:** Piotr Cofta, Damian Ledziński, Sandra Śmigiel, Marta Gackowska

**Affiliations:** Faculty of Telecommunications, Computer Science and Technology, UTP University of Science and Technology, 85-796 Bydgoszcz, Poland; piotr.cofta@utp.edu.pl (P.C.); damian.ledzinski@utp.edu.pl (D.L.); sandra.smigiel@utp.edu.pl (S.Ś.)

**Keywords:** entropy, cross-entropy, drones, swarms, robustness

## Abstract

Due to their growing number and increasing autonomy, drones and drone swarms are equipped with sophisticated algorithms that help them achieve mission objectives. Such algorithms vary in their quality such that their comparison requires a metric that would allow for their correct assessment. The novelty of this paper lies in analysing, defining and applying the construct of cross-entropy, known from thermodynamics and information theory, to swarms. It can be used as a synthetic measure of the robustness of algorithms that can control swarms in the case of obstacles and unforeseen problems. Based on this, robustness may be an important aspect of the overall quality. This paper presents the necessary formalisation and applies it to a few examples, based on generalised unexpected behaviour and the results of collision avoidance algorithms used to react to obstacles.

## 1. Introduction

The development of drones and their swarms will eventually lead to crowded skies, particularly in urban environments. The safety of such environments depends on the way the behaviour of swarms is organised, including the need to keep them within allocated airspace. Furthermore, an appropriate organisation of swarms may have a positive impact on the fulfilment of their missions while requiring limited use of resources and minimising the impact on the environment.

However, the physicality of flight means that the swarm is always affected by some level of disorganisation, whether it is caused by changeable weather or unanticipated objects crossing the flight path. A certain level of disorganisation can be managed but excessive disorganisation may lead to significant damage. It is possible to calculate an acceptable disorganisation profile as a part of mission risk management.

This paper proposes the use of cross-entropy as a metric of the robustness of the swarm control algorithm, where the swarm is treated as a Shannon stochastic information source that is optimised for the acceptable level of disorganisation. Knowing the divergence from the acceptable, referential entropy will help control missions to avoid unacceptable levels of disorganisation and to compare mission control algorithms to identify those that prevent excessive disorganisation. This paper presents research that provides a proposition and some cases to support it. Further work is planned to verify and apply this theory to actual swarms of drones.

This paper starts with a brief note on terminology and goes on to introduce the model of the swarm. Further, the necessary formalisation with brief examples and discussion is presented. Then, an extended example is presented, as well as an overview of some related works with conclusions.

## 2. Terminology

A drone is a general term related to unmanned vehicles of various kinds [1], whether they be remotely operated or autonomous. For this paper, it is beneficial to primarily think of drones in terms of popular multirotor unmanned aerial vehicles, such as quadcopters. A swarm is a collection of drones under a single management system, occupying a certain space, interacting with each other, and pursuing their collective objective while avoiding collisions (we intentionally exclude swarms whose intention is to engage in a collision). A mission is any time during which drones in a swarm move to a specific target while performing the desired trajectory. A mission should be realised optimally and safely. A mission can be performed automatically, semi-automatically or autonomously, depending on the management methods.

## 3. Model

Let us consider a swarm of drones $D=\left\{d_{i};i=1,\ldots ,k\right\}$ that execute mission $M=\left\{t_{j};\;j=1,\ldots ,m;\;t_{j+1}>t_{j}\right\}$, which is defined as an ordered, evenly spaced, sequence of moments in time. To accomplish the mission, drones must progress through a series of respective states, e.g., follow certain trajectories. As long as drones follow their planned trajectories, the swarm is considered to be organised. Unexpected events, such as changes in the weather or the intrusion of objects into flight paths, make the swarm diverge and introduce some degree of disorganisation to the otherwise organised structure of a swarm.

The swarm can withstand disorganisation up to a certain level. While such a level can be defined in different ways, here it is described by an overall disorganisation “mass”, where each divergence contributes to such a mass. Events of low-impact divergences (e.g., being slightly off course) have a small contribution, whereas events of high-impact divergences (e.g., leaving the allocated perimeter) have a large contribution. Certain substitutions are possible, e.g., a few low-impact events can be considered to be the equivalent of a single high-impact one.

Based on the relative occurrence of different events, it is possible to construct the acceptable probability of events such that low-impact events can appear more frequently than high-impact ones. Note that some impact is unavoidable, there is no state free from some disorganisation. Considering that there is a finite precision, the state of being perfectly on course can be determined even if such a state is not free from some impact.

As drones report their behaviour through events, it is possible to determine the difference between the acceptable probability distribution of events and the actual one to find out whether the mission flies at low or high levels of disorganisation.

## 4. Formalisation

Let us assume that there is a set of *n* classes of possible states, each reflecting a certain level of divergence such that at any time interval, the drone can report one of *n* possible events. Those classes (and associated events) are identified as $C=\left\{c_{i},\;i=1,\ldots ,n\right\}$. At regular intervals throughout the mission, each drone communicates the class they are currently in. With every class, there is an associated relative impact factor on disorganisation, $F=\left\{f_{i},\;i=1,\ldots ,n\right\}$. The assessment of such an impact can be provided, e.g., by the analysis of previous missions in a way similar to risk assessment.

From the relationship between various impact factors, it is possible to calculate the normalised discrete probability distribution *Q*, where events from classes associated with the lower impact factor are granted a higher probability of occurrence. For example:

(1) $$Q=\left\{q_{i}\;;\;i=1,\ldots ,\;n\right\};q_{i}=\frac{1}{f_{i}\times \sum_{j=1,\ldots ,n}\frac{1}{f_{j}}}.$$

It is also possible to construct *Q* using other methods, e.g., by sampling previous events in a manner that is often used in risk management. As high-impact events also tend to be low probability events, efficient sampling may be based on importance and may even internally employ cross-entropy to determine *Q* [2].

The distribution *Q* describes the referential probability distribution when the overall disorganisation is still at an acceptable level. This distribution is associated with a referential entropy, i.e., a level of disorganisation that does not disrupt the mission. This referential entropy can be calculated using Shannon’s equation [3]:

(2) $$H\left(q\right)=-\sum_{i=1,\ldots ,n}q_{i}\times \log\left(q_{i}\right).$$

As drones communicate signals, it is possible to determine the probability distribution of receiving various classes of signals, $P=\left\{p_{i},\;i=1,\ldots ,n\right\}$. Based on this, the cross-entropy between the observed and the referential entropy can be calculated using [4]:

(3) $$H\left(p,q\right)=-\sum_{i=1,\ldots ,n}p\left(x_{i}\right)\times \log\left(q\left(x_{i}\right)\right).$$

This cross-entropy can be interpreted as a measure of disorganisation of the swarm relative to the referential one. Note that the cross-entropy can be both smaller and larger than the referential one, indicating situations of (acceptable) low disorganisation and of high (potentially unacceptable) disorganisation, respectively.

For clarification, the levels of entropy that are above the referential one represent an increased risk to the mission but do not necessarily signify its failure. In practice, the levels below the referential one may allow the swarm to continue its operation in a fully automatic or even autonomous manner, while levels higher than the referential one may call for an operator’s action.

For example, let us consider a swarm that can emit five classes of signals that represent five real-life situations, namely *c*_1_: the drone is on course, *c*_2_: the drone is slightly off course but not disturbing other drones, *c*_3_: the drone is within the limits of the swarm but it is disturbing other drones, *c*_4_: the drone has left the perimeter of the swarm and *c*_5_: the drone has lost contact with the swarm.

The impact of various classes on the disorganisation has been determined and shown in Table 1. Such an impact can be associated, e.g., with the mission average delays, energy usage, or other risk factors. Note that it is only the relative impact that is important, not the absolute values.

This implies the following (Table 2) probability distribution (all values rounded).

The referential entropy calculated according to the equation above is approx. 0.090. Therefore, if the actual entropy of the swarm is below this value, the swarm can be characterised as having low disorganisation, while higher values indicate an extent of disorganisation that may unacceptably increase the risk to the mission.

Case 1: Let us consider a situation where the mission has been disturbed such that several drones left their planned trajectories. The observed distribution of events is as shown in Table 3:

For this case, the cross-entropy is approx. 1.840, much higher than the referential one. This indicates an increase in disorganisation and an increased risk.

Case 2: Let us consider the same swarm flying a very quiet mission where the drones fly exactly as planned all the time. The observed distribution *P* has the form of the Kronecker delta (Table 4).

In this case, the cross-entropy is approx. 0.016, lower than the referential one. This indicates that this mission is well organised.

## 5. Continuous and Mixed Probability Distributions

As the size and the density of swarms grow, it is reasonable to consider a continuous case where both probability distributions *P* and *Q* are continuous functions over some support *X*. For example, probability distribution *Q* may be derived from a continuous function linking the distance from the correct state with the impact on disorganisation. Alternatively, distribution *P* can be a continuous estimate of the actual discrete distribution.

(4) $$H\left(p,q\right)=-\int_{x\in X}P\left(x\right)\times \log Q\left(x\right)\;dx$$

Situations of mixed distributions, where *Q* is continuous while *p* is discrete, are of some practical use. For example, the drone management system may define the continuous impact function that links the distance between the expected and the actual state to the extent of the impact, resulting in a continuous distribution of *Q*. Meanwhile *p* can be discrete, as it is being calculated from events observed during the swarm mission.

For such situations, the cross-entropy can be determined as follows, where *Q*(*x*) is the value of the probability distribution function calculated using the impact function for given value *x*:

(5) $$H\left(p,q\right)=-\sum_{x\in X}p\left(x\right)\times \log\left(Q\left(x\right)\right).$$

An alternative approach may require the use of the fixed-width quantisation of functions over the support in the form of an LDDP (limiting density of discrete points). This may introduce the need for correction of the quantisation error [5,6]. Note that the use of cross-entropy in this paper is a comparative one: it is more interesting to compare two values than present their correctness. Consequently, both approaches may provide a solution.

## 6. Discussion

### 6.1. Advantages of Cross-Entropy

The difference between the desired and the actual state of drones can be expressed through various loss metrics, specifically using the mean squared error (MSE) or through cross-entropy. The choice of cross-entropy over MSE comes from the intended purpose of the loss metric, namely to improve the control algorithm for the swarm of drones. Compared to MSE, cross-entropy stresses the small differences from the referential level of entropy, i.e., cross-entropy allows for better differentiation between what is acceptable (in terms of disorganisation) and what is excessive.

The problem of drones diverging from their intended trajectories is mostly seen as a control problem with an impact on energy loss. Whatever method is used to manage the swarm, it has to determine its situation and undertake control measures to revert drones to their routes. Thus, the key determinants of the extent of the divergence are the control precision and energy loss. Out of these two loss metrics, cross-entropy that is a better estimator for the required additional amount of information.

From the perspective of control, small differences must not be neglected, as they still require actions to be taken and the swarm control algorithm must be adjusted to react to them without over-reacting. In contrast, when the difference is large, the extent of this difference gradually matters less from the perspective of control, as only some coarse actions have to be taken.

Similarly, from the perspective of energy consumption and owing to the inertia of the drone, small manoeuvres are relatively expensive (per the unit of distance), while large manoeuvres can be inexpensive. Thus, the cross-entropy seems to be a better estimator for both the control overhead and the use of energy.

### 6.2. Coding Scheme

Cross-entropy is susceptible to the choice of the coding scheme (i.e., the distribution *q*). Specifically, *q* must anticipate all possible classes of signals by assigning them certain non-zero probabilities over all the support of *p*. If such a distribution is derived from the impact factor, then no signal is allowed to be free from the impact. Otherwise, if the swarm is in situations not anticipated by *q* (i.e., where the distribution is either undefined or zero), the information content of such signals is undefined or infinite.

The construction of an appropriate *q* can be done using different methods; this paper does not assume that the impact factor is the only appropriate one. The impact factor is conceptually similar to methods used in machine learning, where there are weighted penalties for misclassification [7]. The authors’ choice of a method for the determination of the probability distribution was affected by two factors. First, the probability distribution forms the common denominator for various methods to determine *q*. Second, the problem of controlling the swarm is a signalling and information problem; therefore, adherence to the cross-entropy origin was preferential.

There is a further similarity between the problem of constructing *q* and machine learning where a training set does not have samples of some classes, leading to difficulties in calculating the cross-entropy. This is improved by the recommendation to include samples for all classes. Similarly, this paper contains a restriction that there is no class without a certain impact. Note that, formally, this approach is justified: in real life, the class of “being perfectly on course” cannot be described as a Dirac delta due to measurement imprecision. Hence, it must always contain some range of low-impact behaviours.

The problem of assigning an appropriate probability to events that are only anticipated or that are rare is known, with some propositions applying cross-entropy to optimise the importance of sampling [2]. This proposition does not improve on them, as it only assumes that *q* can be determined. The proposed use of a real-life impact function closely links the proposed metric with actual costs and benefits of various behaviours, without claiming any particular shape of the impact function.

### 6.3. Referential Entropy

This model uses a referential probability distribution and its referential entropy to calculate the cross-entropy. It makes cross-entropy both positive and negative, depending on whether the actual distribution of signals indicates that the level of disorganisation is higher or lower than the referential one. Consequently, in a way that is different than in other applications of cross-entropy, there is no minimum entropy level that the cross-entropy will always be higher than, even though such cross-entropy can be used to judge (and to optimise) the swarm management algorithm.

There is an underlying assumption that the swarm can take certain levels of disorganisation such that not every localised disorganisation has an immediate impact on the swarm. Specifically, that imprecision in measurements may not allow us to confirm whether any drone is actually in the desired state. Therefore, the algorithm quality should not be judged by absolute perfection but rather by staying on the safe side of the referential level of disorganisation.

This reasoning means that the Kullback–Leibler (K-L) divergence is not applicable to this case. K-L divergence provides an estimate of the difference between the entropy of the actual distribution and the cross-entropy. In this case, it is at its minimum when *p* = *q*, i.e., when the swarm is disorganised exactly in an acceptable way. Whether the actual distribution indicates more or less disorder than the swarm can bear, the K-L divergence will grow. Still, K-L divergence may be a useful metric of the distance from the referential disorganisation that helps design control algorithms.

## 7. Extended Example

Collision avoidance is an example of a process that temporarily increases the disorganisation of the swarm. That is, in the presence of a disturbance, drones have to veer off course and break the formation. In such situations, they find themselves at locations that are less expected. Intuitively, the entropy of a swarm should increase. However, such an increase should only be temporary. In the absence of continuous disturbances, drones should return to the desired paths to continue their mission. As the drones again appear at the expected locations, the entropy of the swarm should decrease. Thus, the managed swarm should be perceived as an entropy-minimisation device, i.e., its entropy will increase only to the extent required to overcome an obstacle and will decrease once the obstacle is removed. The quality of such a strategy is reflected by the level of entropy carried by the swarm throughout the mission.

Collision avoidance by unmanned aerial vehicles can be implemented in many ways. Gong et al. [8] used a gradient-based collision avoidance algorithm for the control of multi-agent formations. The algorithm uses a consensus theory and a graph theory applied to three topologies. This method uses two circular zones that define distances from adjacent objects, whether they be obstacles or neighbours, to define prohibited zones.

This section makes use of the collision avoidance algorithm developed by Cofta et al. [9] that bears a superficial similarity to the one described above. The algorithm itself is inspired by the physics of repulsive forces (see, e.g., [10]), thus algorithmically replicating the exclusivity. The algorithm is run by each drone independently for at least at fixed time intervals and always planning for the interval ahead. It considers the location and movement of neighbouring drones. If some drones are nearby, then the algorithm alters the paths of drones to avoid close encounters; otherwise, they attempt to continue their original mission. The equations that define the algorithm are as follows:

(6) $${\vec{p}}_{z}={\vec{p}}_{m}+\tau *{\vec{p}}_{u},$$

(7) $${\vec{p}}_{i}=\vec{\left(x_{i},x_{j}\right)},$$

(8) $$i\in N↔\left|{\vec{p}}_{i}\right|\leq R\;\mathrm{and}\;i\neq j,$$

(9) $${\vec{u}}_{i}=\frac{{\vec{p}}_{i}}{\left|{\vec{p}}_{i}\right|},$$

(10) $${\vec{p}}_{u}=\underset{i\in N}{\sum }{\vec{u}}_{i}{\left(R-\left|{\vec{p}}_{i}\right|\right)}^{Q},$$

where:

${\vec{p}}_{z}$—vector that the drone will adopt for the next time interval.
${\vec{p}}_{m}$—mission vector, i.e., the vector that the drone should have adopted to continue the mission; this vector is calculated to achieve the objective of the mission in the current situation if it were ignoring obstacles.
${\vec{p}}_{u}$—escape vector that should be adopted to escape close encounters with neighbours disregarding the mission.
$x_{j}$—drone’s location.
$x_{i\;}$—the location of the neighbour i.
${\vec{u}}_{i}$—unit vector of ${\vec{p}}_{i\;}$.
*N*—the set of neighbours within radius *R*.
*R*—radius within which neighbours are of interest (in this simulation *R* = 25 m).
*Q* and *τ*—parameter constants (in this simulation *Q* = 1 and *τ* = 1).

The following examples were developed using the simulation software created by the authors. The probability function used in those examples directly relates the distance between the actual and expected locations of the drone. It is defined as the cumulative distribution function of the lambda distribution. Consequently, the probability distribution *Q* is a lambda function $Q\left(x\right)=\lambda e^{-\lambda x};\;\lambda =1$, with a referential entropy of 1. As the *p* distribution is a discrete one, the mixed version of cross-entropy is used, where the sum is run over the product of the discrete probability distribution *p* and the point value of *Q*(*x*).

This approach allows for an introduction of “momentary” entropy, where the distribution *p* takes on the form of the Kronecker delta. This allows for analysing changes in the entropy over time in response to obstacles for an individual drone or a swarm of them. While the “momentary” entropy is not intended to be used as a metric of robustness, it is a useful tool to illustrate and analyse the behaviour of drones and swarms.

Video recordings of simulations related to the extended example are included as Supplementary Materials.

### 7.1. Close Encounter

**Figure 1:**
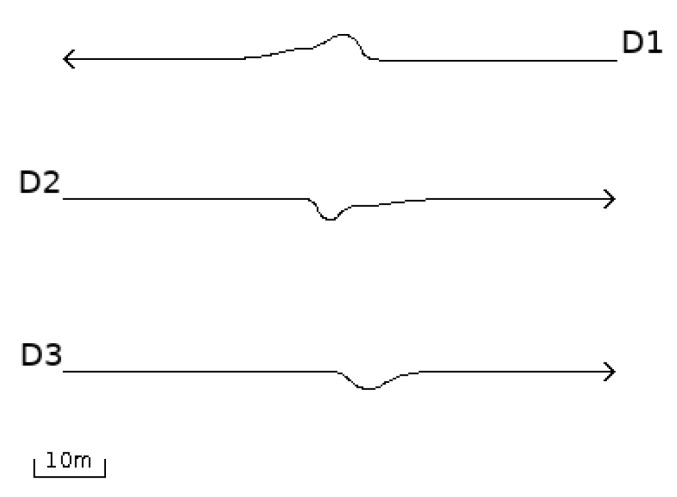
Close encounter of drones.

Figure 1 shows the trajectory of the flight of a swarm D that consists of three drones. D_2_ and D_3_ fly to the east, while D_1_ flies to the west. Their trajectories were close enough such that the collision avoidance algorithm was triggered for all of them.

**Figure 2:**
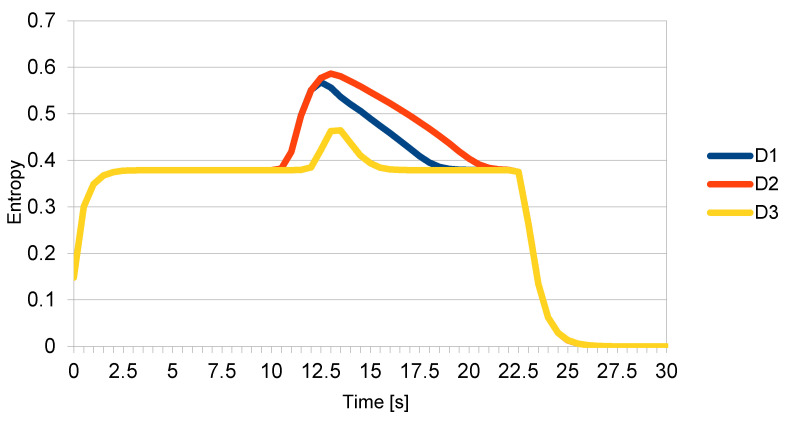
Momentary entropy of each drone as a function of time.

When the two topmost drones got too close to each other, they both veered off their courses, as expected. However, this caused the central drone to move closer to the lower one, triggering its algorithm as well. Eventually, all drones moved away from their courses. Once the distance became safe again, they returned to their original courses. Figure 2 shows the momentary entropy of each drone as a function of time.

### 7.2. Grid Formation

**Figure 3:**
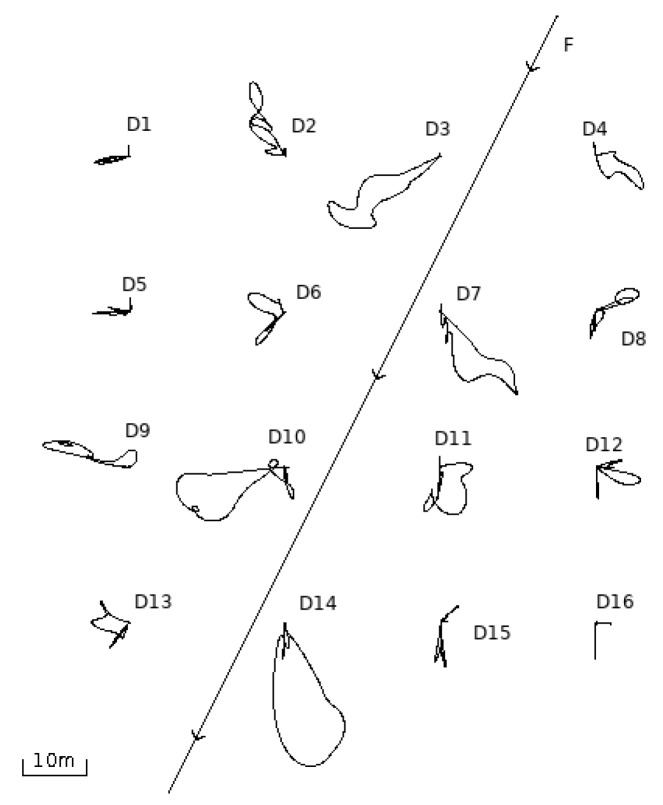
Sixteen-drone swarm with an initial grid formation. The swarm avoids the intruder (straight line) before returning to the grid formation via the paths shown.

Figure 3 shows a sixteen-drone swarm with drones stationary in a grid formation. The objective of their mission was to remain in this formation and at their current positions. The simulation depicted a situation where there was an intruder drone that passed through the swarm, paying no attention to other drones, to the extent of potentially colliding with them. However, each drone in the swarm was paying attention to every other drone, including the intruder, executing the collision avoidance algorithm. Note that the path of the intruder was straight, while drones from the swarm gave way. Once the intruder passed, each drone returned to its assumed position in the formation. Because the formation was relatively dense, the movement of one drone affected its neighbours.

**Figure 4:**
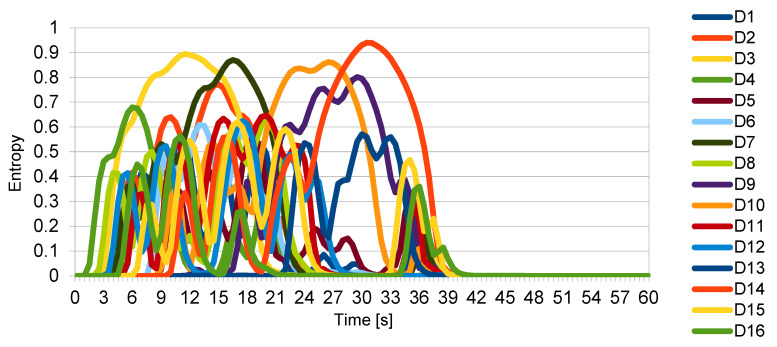
Momentary entropy of drones within the swarm.

**Figure 5:**
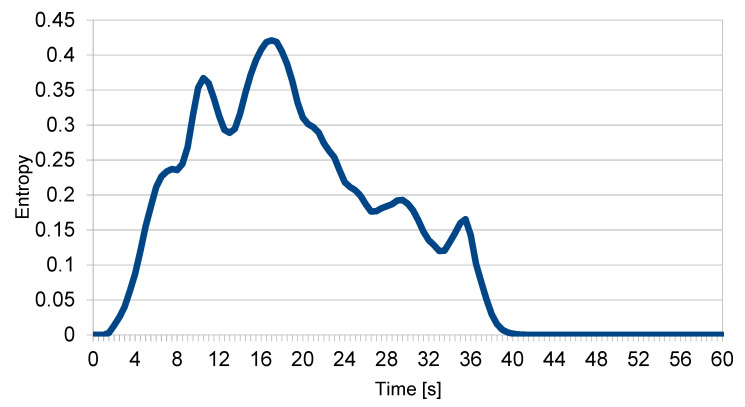
Momentary entropy of the swarm.

Figure 4 shows the change in the momentary entropy over time of particular drones from the swarm. Figure 5 shows the total momentary entropy of the swarm over time. As expected, the appearance of the intruder initially increased the entropy but once the intruder left the swarm, it eventually returned to just above zero.

Note that the momentary entropy assumed the value of −log(*Q*(*x*)), i.e., it could reach any positive value. The change in entropy over time, as shown in Figure 5, was calculated by including events from all drones for a given moment in time. As all drones reported events at regular intervals, this made the values presented in Figure 5 the average of the respective values from Figure 4.

Note that neither the momentary entropy of the drones nor the momentary entropy of the swarm ever exceeded the value of the referential entropy, which means that the level of disorganisation was acceptable. The entropy of the whole swarm, calculated for the whole passage of the intruder, was approx. 0.14. Again, this indicates that, despite the disturbances, the swarm was reasonably organised and that the mission itself was not endangered.

## 8. Related Works

Cross-entropy is a measure of a discrepancy between two probability distributions [4]. It is used widely beyond the theory of information, e.g., as an objective function for the optimisation of traffic flow [11] or in a particle swarm optimisation [12,13]. It is also used in machine learning as a loss function for the training set of neural networks [14] or to improve the clustering of data [15]. Further, it is used in robotics for the optimisation of controllers based on fuzzy logic [16,17]. Regarding the social sciences, it can also be used to explain complex global behaviours [18] and can be used in swarm intelligence [19].

Swarms and their self-organisation borrows much from the observation of bees [20], locusts [21], birds and fish [22,23,24,25]. The close resemblance between unmanned aircraft and insects or animals has been researched [26], specifically in terms of collision avoidance and in collaborative intelligence [27], while Can et al. [28] applies the rules of particle physics to swarms.

In those diverse areas, entropy is defined by re-applying Shannon’s formula to various forms of grouping. For example, Folino and Forestiero [29], inspired by Van Dyke Parunak and Brueckner [30], demonstrates how entropy can be used to assess the properties of self-organising flocking algorithms by observing changes in entropy resulting from coupling organised and disorganised systems.

The application of cross-entropy to the process of training neural networks bears some resemblance to the problem discussed here, specifically for discrete distributions. Cross-entropy is one of the standard loss functions, specifically regarding multi-label classification [31]. Such a loss function can be extended to include some penalties for mislabelling [32,33], making it attractive for some real-world cases where misclassification should be penalised [6].

Particle swarm optimisation (PSO) [34] can use entropy for the simulated set of states (“particles”) (EA-PSO) [12], and then it may apply cross-entropy in the meta-optimisation of the search space. Various modifications and extensions exist, such as memetic based [13], niche strategy [35], or clustering [36]. The evolutionary approach is used in Hu et al. [37], while Zhang et al. [38] employs direct competition.

PSO may prematurely converge to local optima since the best performing particle attracts the remaining ones. Therefore, the problem of diversity management (i.e., having particles exploring different alternatives beyond the local optimum) is important. Entropy is used in a way inspired by Shannon to manage the extent of diversity at the swarm level, combined with the optimum-seeking behaviour of particles at the local level.

Cross-entropy is used by PSO (among others) as a way to meta-optimise the solution space. For example, Yin [39] applies cross-entropy minimisation to determine the optimum threshold in image segmentation by comparing the probability distribution of the original image and the one after a threshold has been applied.

## 9. Conclusions

This paper proposes the use of cross-entropy as a metric for the quality of algorithms that manage swarms of drones. It reflects the extent of disorganisation of the swarm throughout its mission, where such disorganisation should be always minimised as much as possible. This cross-entropy is calculated relative to the referential probability distribution that is constructed out of the real-world impact that various events may have on the swarm.

Initial simulations demonstrated the viability of cross-entropy as a metric and allowed for distinguishing between missions with low and high levels of disorganisation. This is a work in progress. The authors plan to run both simulations and field experiments to determine the practical usefulness of the proposed metric under various flight conditions.

## Figures and Tables

**Table 1:** Relative impact of classes of events.

*c*_1_	*c*_2_	*c*_3_	*c*_4_	*c*_5_
0.01	1.0	20.0	50.0	100.0

**Table 2:** Probability distribution for classes of events.

*q*_1_	*q*_2_	*q*_3_	*q*_4_	*q*_5_
0.9893	0.0990	0.0005	0.0002	0.0001

**Table 3:** Probability distribution for significant disturbances.

*p*_1_	*p*_2_	*p*_3_	*p*_4_	*p*_5_
0.8	0.1	0.06	0.03	0.01

**Table 4:** Probability distribution for the undisturbed mission.

*p*_1_	*p*_2_	*p*_3_	*p*_4_	*p*_5_
1.0	0.0	0.0	0.0	0.0

## Supplementary Materials

The following are available online at https://www.mdpi.com/1099-4300/22/6/597/s1, Video S1: Grid, Video S2: Grid (debug), Video S3: Simple, Video S4: Simple (debug).

## References

[1] Parlin K., Alam M.M., Le Moullec Y. Jamming of UAV remote control systems using software defined radio. Proceedings of the 2018 International Conference on Military Communications and Information Systems (ICMCIS).

[2] Ridden A., Rubinstein R. (2007). Minimum Cross-entropy Methods for Rare-event Simulation. Simulation.

[3] Shannon C.E. (1948). A mathematical theory of communication. Bell Syst. Tech. J..

[4] Rao C.R., Alladi K., Klauder J., Rao C. (2010). Entropy and Cross Entropy: Characterizations and Applications. The Legacy of Alladi Ramakrishnan in the Mathematical Sciences.

[5] Jaynes E.T., Ford K. (1963). Information Theory and Statistical Mechanics. Statistical Physics (PDF).

[6] Jaynes E.T. (1968). Prior Probabilities. IEEE Trans. Syst. Sci. Cybern..

[7] Mahdy M. (2018). Weighted Entropy Measure: A New Measure of Information with its Properties in Reliability Theory and Stochastic Orders. J. Stat. Theory Appl..

[8] Gong Q., Wang C., Qi Z., Ding Z. Gradient-based collision avoidance algorithm for second-order multi-agent formation control. Proceedings of the 2017 36th Chinese Control Conference (CCC).

[9] Cofta P., Ledziński D., Śmigiel S., Gackowska M., Jerks N. (2019). Swarm Drone Communication & Collision Avoidance. Patent Application.

[10] Geiger H., Marsden E. (1909). On a Diffuse Reflection of the α-Particles. Proc. R. Soc. A.

[11] Ma T.-Y., Lebacque J.-P. (2009). A Cross Entropy Based Multi-Agent Approach to Traffic Assignment Problems. Traffic Granul. Flow.

[12] Guo W., Zhu L., Wang L., Wu Q., Kong F. (2019). An Entropy-Assisted Particle Swarm Optimizer for Large-Scale Optimization Problem. Mathematics.

[13] Petalas Y.G., Parsopoulos K.E., Vrahatis M.N. Entropy–Based Memetic Particle Swarm Optimization for Computing Periodic Orbits of Nonlinear Mappings. Proceedings of the 2007 IEEE Congress on Evolutionary Computation.

[14] Aurelio Y.S., de Almeida G.M., de Castro C.L., Braga A.P. (2019). Learning from Imbalanced Data Sets with Weighted Cross-Entropy Function. Neural Process. Lett..

[15] Liu B., Pan J., McKay R.I. (2009). Entropy-based metrics in swarm clustering. Int. J. Intell. Syst..

[16] Anisimov D.N., Dang T.S., Banerjee S., Mai T.A. (2017). Design and implementation of fuzzy-PD controller based on relation models: A cross-entropy optimization approach. Eur. Phys. J. Spec. Top..

[17] Olivares-Mendez M.A., Mejias L., Campoy P., Mellado-Bataller I. (2012). Cross-Entropy Optimization for Scaling Factors of a Fuzzy Controller: A See-and-Avoid Approach for Unmanned Aerial Systems. J. Intell. Robot. Syst..

[18] Kordon A.K. (2010). Swarm Intelligence: The Benefits of Swarms. Applying Computational Intelligence.

[19] Blum C., Li X., Blum C., Merkle D. (2008). Swarm Intelligence in Optimization. Swarm Intelligence. Natural Computing Series.

[20] Cully S.M., Seeley T.D. (2004). Self-assemblage formation in a social insect: The protective curtain of a honey bee swarm. Insectes Sociaux.

[21] Kurdi H.A., Aloboud E., Alalwan M., Alhassan S., Alotaibi E., Bautista G., How J.P. (2018). Autonomous task allocation for multi-UAV systems based on the locust elastic behavior. Appl. Soft Comput..

[22] Parrish J.K., Viscido S.V., Grünbaum D. (2002). Self-Organized Fish Schools: An Examination of Emergent Properties. Biol. Bull..

[23] Okubo A. (1986). Dynamical aspects of animal grouping: Swarms, schools, flocks, and herds. Adv. Biophys..

[24] Toner J., Tu Y. (1998). Flocks, herds, and schools: A quantitative theory of flocking. Phys. Rev..

[25] Reynolds C.W. Flocks, herds and schools: A distributed behavioral model. Proceedings of the 14th Annual Conference on Computer Graphics and Interactive Techniques (SIGGRAPH 1987), Association for Computing Machinery.

[26] Janson S., Middendorf M., Beekman M. (2005). Honeybee swarms: How do scouts guide a swarm of uninformed bees?. Anim. Behav..

[27] Vásárhelyi G., Virágh C., Somorjai G., Nepusz T., Eiben A.E., Vicsek T. (2018). Optimized flocking of autonomous drones in confined environments. Sci. Robot..

[28] Can C.F., Bayram Ç., Toksoy A.K., Avsar H., Ozdemir S. (2005). Characterization of Swarm Beahavior through Pairwise Interactions by Tsallis Entropy.

[29] Folino G., Forestiero A., González J.R., Pelta D.A., Cruz C., Terrazas G., Krasnogor N. (2010). Using Entropy for Evaluating Swarm Intelligence Algorithms. Nature Inspired Cooperative Strategies for Optimization (NICSO 2010). Studies in Computational Intelligence.

[30] Van Dyke Parunak H., Brueckner S. (2001). Entropy and self-organization in multi-agent systems. Proceedings of the Fifth International Conference on Autonomous Agents (AGENTS 2001).

[31] Dong Q., Zhu X., Gong S. (2019). Single-Label Multi-Class Image Classification by Deep Logistic Regression. Proc. Conf. Artif. Intell..

[32] Rengasamy D., Jafari M., Rothwell B., Chen X., Figueredo G.P. (2020). Deep Learning with Dynamically Weighted Loss Function for Sensor-Based Prognostics and Health Management. Sensors.

[33] Ho Y., Wookey S. (2020). The Real-World-Weight Cross-Entropy Loss Function: Modeling the Costs of Mislabeling. IEEE Access.

[34] Kennedy J., Eberhart R.C. (1995). Particle swarm optimization. Proceedings of the IEEE International Conference on Neural Networks.

[35] Li D., Guo W., Wang L., Tan Y., Shi Y., Niu B. (2019). Niching Particle Swarm Optimizer with Entropy-Based Exploration Strategy for Global Optimization. Advances in Swarm Intelligence. ICSI 2019. Lecture Notes in Computer Science.

[36] Çomak E. (2015). A modified particle swarm optimization algorithm using Renyi entropy-based clustering. Neural Comput. Appl..

[37] Hu W., Hu J., Zhang X., Lei J., Wang F.L., Deng H., Miao D. (2012). The Improved Particle Swarm Optimization Based on Swarm Distribution Characteristics. Artificial Intelligence and Computational Intelligence. AICI 2012. Lecture Notes in Computer Science.

[38] Zhang W.-X., Chen W.-N., Zhang J. A dynamic competitive swarm optimizer based-on entropy for large scale optimization. Proceedings of the 2016 Eighth International Conference on Advanced Computational Intelligence (ICACI).

[39] Yin P.-Y. (2007). Multilevel minimum cross entropy threshold selection based on particle swarm optimization. Appl. Math. Comput..

